# Description of Chloramphenicol Resistant *Kineococcus rubinsiae* sp. nov. Isolated From a Spacecraft Assembly Facility

**DOI:** 10.3389/fmicb.2020.01957

**Published:** 2020-08-18

**Authors:** Snehit Mhatre, Nitin K. Singh, Jason M. Wood, Ceth W. Parker, Rüdiger Pukall, Susanne Verbarg, Brian J. Tindall, Meina Neumann-Schaal, Kasthuri Venkateswaran

**Affiliations:** ^1^Biotechnology and Planetary Protection Group, Jet Propulsion Laboratory, California Institute of Technology, Pasadena, CA, United States; ^2^Leibniz-Institute DSMZ-German Collection of Microorganisms and Cell Cultures, Braunschweig, Germany

**Keywords:** antibiotic resistant bacteria, genome, cleanroom, spacecraft assembly facility, *Kineococcus rubinsiae*

## Abstract

A Gram-positive, coccoid, motile, aerobic bacterium, designated strain B12^T^ was isolated from a Jet Propulsion Laboratory spacecraft assembly cleanroom, Pasadena, CA, United States. Strain B12^T^ was resistant to chloramphenicol (100 μg/mL), and is a relatively slow grower (3–5 days optimal). Strain B12^T^ was found to grow optimally at 28 to 32°C, pH 7 to 8, and 0.5% NaCl. Fatty acid methyl ester analysis showed that the major fatty acid of the strain B12^T^ was anteiso C_15__:__0_ (66.3%), which is also produced by other *Kineococcus* species. However, arachidonic acid (C_20__:__4_ ω6,9,12,16c) was present in strain B12^T^ and *Kineococcus glutinatus* YIM 75677^T^ but absent in all other *Kineococcus* species. 16S rRNA analysis revealed that strain B12^T^ was 97.9% similar to *Kineococcus radiotolerans* and falls within the *Kineococcus* clade. Low 16S rRNA gene sequence similarities (<94%) with other genera in the family *Kineosporiaceae*, including *Angustibacter* (93%), *Kineosporia* (94% to 95%), *Pseudokineococcus* (93%), *Quadrisphaera* (93%), and *Thalassiella* (94%) demonstrated that the strain B12^T^ does not belong to these genera. Phylogenetic analysis of the *gyrB* gene show that all known *Kineococcus* species exhibited <86% sequence similarity with B12^T^. Multi-locus sequence and whole genome sequence analyses confirmed that B12^T^ clades with other *Kineococcus* species. Average nucleotide identity of strain B12^T^ were 75–78% with other *Kineococcus* species, while values ranged from 72–75% with species from other genera within family *Kineosporiaceae*. Average amino-acid identities were 66–72% with other *Kineococcus* species, while they ranged from 50–58% with species from other genera. The dDDH comparison of strain B12^T^ genome with members of genera *Kineococcus* showed 20–22% similarity, again demonstrating that B12^T^ is distantly related to other members of the genus. Furthermore, analysis of whole proteome deduced from WGS places strain B12^T^ in order *Kineosporiales*, confirming that strain B12^T^ is a novel member of family *Kineosporiaceae*. Based on these analyses and other genome characteristics, strain B12^T^ is assigned to a novel species within the genus *Kineococcus*, and the name *Kineococcus rubinsiae* sp. nov., is proposed. The type strain is B12^T^ (=FJII-L1-CM-PAB2^T^; NRRL B-65556^T^, DSM 110506^T^).

## Introduction

Traditionally, biochemical characteristics (Phillips et al., 2002), chemotaxonomic analyses (Lee et al., 2016), and DNA-DNA hybridization analyses (Satomi et al., 2006) were used to describe novel microbial species, including members belonging to the *Actinobacteria* phylum. Furthermore, fatty acid methyl ester (FAME) (Diogo et al., 1999) and matrix-assisted laser desorption/ionization time of flight (MALDI-TOF) mass spectrometry analyses (Seuylemezian et al., 2018) have been shown to be a useful alternative or adjunct to phenotypic methodologies for the identification of many bacteria. Most of these traditional bacterial identification methods were not feasible for environmental bacteria since traditional databases were dependent on fast growing, clinically important microorganisms. The slow growing *Actinobacteria* tend to secrete biochemical compounds and enzymes differentially in various cultivation media and also depend on specific growth conditions, hence phenotype-based characterization will not resolve to their species.

Identifying bacterial species, despite its eminent practical significance for identification, diagnosis, quarantine, and diversity surveys, remains a very difficult issue to advance. To overcome issues related to potentially unknown cultivation conditions for an organism, genomic sequences have recently been used to unambiguously identify microbial taxa (Singh et al., 2019). Several strategies, including amplicon sequencing of phylogenetic marker genes (16S rRNA, *gyrB*, etc.), multi-locus sequencing analysis (MLSA), and whole genome sequencing (WGS) were implemented to differentiate hard to identify microbial species (Venkateswaran et al., 2017; Singh et al., 2019). Furthermore, minimal standards for the use of genome data for the taxonomy of prokaryotes were proposed (Konstantinidis et al., 2017; Chun et al., 2018). Phylogenetically indistinguishable members detected using 16S rRNA gene sequence analysis in the *Bacillus cereus* group (La Duc et al., 2004) were differentiated using MLSA analysis (Helgason et al., 2000). Genomics now offers novel insights into intra-species/genus diversity and the potential for emergence of a more soundly based system (Chun et al., 2018). Konstantinidis et al. (2006) analyzed several bacterial strains and revealed what is actually encompassed in a species according to the current standards, in terms of WGS and gene-content diversity. Konstantinidis et al. (2006) and Varghese et al. (2015) reported that the genome-wide average nucleotide identity (ANI) metric, which is widely acknowledged as a robust measure of genomic relatedness, should be combined with the digital DNA-DNA hybridization (dDDH) values between two genomes to accurately define microbial taxa. The average amino-acid identity (AAI) metric has also been proposed as a standard for high-quality descriptions of microbial taxa (Konstantinidis et al., 2017). The ANI, AAI, and dDDH values were used to address the novelty of a strain isolated during this study for bacterial species classification and as the primary guide for new taxonomic genus/species assignment, supplemented by the traditional polyphasic approach.

At the time of writing, the order *Kineosporiales* of phylum *Actinobacteria* is comprised of six genera: *Angustibacter* (Ko and Lee, 2017), *Kineococcus* (Phillips et al., 2002), *Kineosporia* (Kudo et al., 1998), *Pseudokineococcus* (Jurado et al., 2011), *Quadrisphaera* (Maszenan et al., 2005), and *Thalassiella* (Lee et al., 2016). *Actinobacteria* represent a very primitive lineage of prokaryotes known to be ubiquitous, slow growing, and is comprised of aerobes and anaerobes, are motile or non-motile, spore formers and non-spore formers, and have a high GC content in genomic DNA (Gao and Gupta, 2005). *Actinobacteria* also play an important role in several biological processes, including bioremediation (Chen et al., 2015), bio-weathering (Cockell et al., 2013), biogeochemical cycles, and promoting plant growth (Palaniyandi et al., 2013). Additionally, they produce a vast array of industrially important bioactive compounds like antibiotics, enzyme inhibitors, anti-inflammatory compounds, and anti-tumor compounds (Jiang et al., 2017). Actinobacteria are known to occur in many extreme environments mainly characterized by extremes of pH, temperature, salinity, radiation, or low levels of nutrients and carbon sources (Zenova et al., 2011). Owing to their diverse metabolism and physiology, *Actinobacteria* can survive hostile and unfavorable conditions (Mohammadipanah and Wink, 2016).

In this communication, an aerobic, coccoid-shaped, and Gram-positive bacterium that was isolated from the National Aeronautics and Space Administration (NASA) Jet Propulsion Laboratory (JPL) spacecraft assembly facility (SAF) cleanroom surface is described. Bacterial classification of the novel species is presented to describe *Kineococcus rubinsiae* sp. nov. that belongs to the family *Kineosporiaceae* of phylum *Actinobacteria*. The whole-genome sequence (WGS) and annotation of *K. rubinsiae* sp. nov., are documented in classifying it as a new member of the family *Kineosporiaceae.*

## Materials and Methods

### Sample Collection and Isolation of Bacteria

Samples were collected from the JPL-SAF, Pasadena, California from 10 locations of 1 m^2^ area using sterile polyester wipes (23 cm × 23 cm; ITW Texwipe, Mahwah, NJ, United States) premoisented with phosphate buffer saline (PBS). Subsequently, each wipe was individually transferred to 200 mL PBS and vortexed at maximum speed for 5 s. The resulting suspension was then concentrated to appropriate volume (∼30-fold) with a concentration pipette CP-150 (Innova Prep, Drexel, MO, United States) using a 0.20 μm hollow fiber polysulfone tip (Kwan et al., 2011). The concentrated samples were then split in two 3 mL aliquots where one aliquot was treated with 100 μg/mL of chloramphenicol (Sigma, St. Louis, MO, United States) and the other aliquot was untreated. The sample amended with chloramphenicol was incubated at 25°C for 24 h and subsequently subjected to downstream processing utilizing both traditional microbiology and molecular biological techniques. At the time the experiments were conducted, the aim was the isolation of novel fungal species thriving in the JPL SAF, and hence a 100 μL suspension of the samples that were treated with and without chloramphenicol was spread onto antibiotic supplemented (chloramphenicol 100 μg/mL) media like potato dextrose agar (PDA) and Dichloran Rose Bengal Chloramphenicol Agar (DRBC) and incubated at 25°C for 5 days. Among several fungal colonies purified, some bacterial colonies also surfaced onto the PDA and DRBC media, and were selected for further characterization. Since the isolation was carried out on antibiotic treated samples, bacteria exhibiting resistance to chloramphenicol were interesting. The bacterial strains resistant to chloramphenicol that exhibited higher 16S rRNA gene similarities (>99%) with already described species were not further studied in this project. However, one of the several bacterial colonies (strain B12^T^) showed 97.9% 16S rRNA gene sequence similarity with its closest neighbor (see below for details). Strain B12^T^ was further assayed for its polyphasic taxonomic and WGS characterization. Distinct colonies of strain B12^T^ were isolated and transferred to fresh PDA medium and subsequently archived in semi-solid R2A media and stored at room temperature as well as in glycerol stock for further characterization.

### Morphological and Phenotypic Characterizations of Strain B12^T^

The cells of strain B12^T^ were fixed for scanning electron microscopy (SEM) by first suspending two separate colonies of the strain B12^T^ in 0.1 M phosphate buffered saline (PBS, pH 7.2; Sigma-Aldrich) in separate 1.5 mL microcentrifuge tubes. In order to obtain B12^T^ cells with decreased amounts of extracellular polysaccharides (EPS), the first tube was vigorously pipetted and then vortexed for 30 s, followed by filtration of the suspension through a 0.2 μm polycarbonate filter membrane on a vacuum manifold. The original tube was washed two more times with 0.1 M PBS, and then passed through the same filter. The filter membrane was then removed and placed into a fresh 1.5 mL microcentrifuge tube. The unbroken-up colony of B12^T^ (with preserved EPS) had excess PBS aliquoted off. The following steps were performed for both samples identically. Suitable aliquots (750 μL) of 2.5% glutaraldehyde in 0.1 M PBS was added to each tube and then incubated in the refrigerator at 4°C for 1 h. Without disturbing the colony or the filter membrane, the majority of the solution was aliquoted from the microcentrifuge tube and replaced with 1 mL of 0.1 M PBS, and returned to the refrigerator for a 10 min. incubation. This wash step was repeated for a total of three times. Next the samples underwent an ethanol (EtOH) dehydration series. The solution was aliquoted out of the microcentrifuge tubes and replaced with an increasing concentration of EtOH diluted in 0.1 M PBS. After each new EtOH solution was added, the tube was incubated in the refrigerator for 10 min. The EtOH concentrations were 50, 70, 80, 90, 95, and 100%. The 100% EtOH was aliquoted out and replaced with fresh 100% EtOH a total of three times, and stored in the refrigerator. The samples then underwent critical point drying in a Tousimis Automegasamdri 915B critical point dryer (Rockville, MD, United States). Samples were then adhered to carbon tape (Ted Pella Inc., Redding, CA, United States) and sputter coated with AuPd using an Anatech Hummer (Sparks, NV, United States) sputter coater. SEM was performed on a FEI Quanta 200F (Themo Fisher, Waltham, MA, United States).

Phenotypic characterization of the strain B12^T^ was performed by following standardized protocols (Jones, 1981). For phenotypic tests, strain B12^T^ was grown in sterile peptone-tryptone-yeast extract-glucose (PTYG; 5 g each per liter) medium incubated at 32°C with pH 7 and 0.5% NaCl, unless otherwise stated. Morphology, size, and pigmentation were observed on PTYG medium after 72 h of incubation. A commercially available kit (BD Difco) was used to determine the Gram-staining status of the strain. Motility was determined by inoculating a loopful of culture into a PTYG broth and incubating it for a period of 72 h. Subsequently, a loopful of broth was tested for motility using a previously established technique (Skerman, 1960). Growth in various temperature conditions (5–45°C) was tested by increasing incubation temperature in increments of 5°C and grown in PTYG broth. Similarly, the pH tolerance (4–10) was tested by adjusting the pH of the PTYG broth with biological buffers (Xu et al., 2005). The NaCl tolerance (0–5%) tests were carried out in 1% sterile peptone broth containing appropriate amounts of NaCl. The carbon substrate utilization profile was carried out as per the BioLog protocol for actinobacteria using GEN III MicroPlate test assay with a Biolog system. The test panel comprises 71 carbon sources with 23 chemical sensitivity assays and thus provides a “Phenotypic Fingerprint” of the tested microorganism. Since B12^T^ cells grown either in tryptic soy broth or Luria broth and washed in buffer before placing in BioLog plates did not exhibit any carbon substrate utilization profile, cells were grown in PTYG medium but such modified growth medium also did not show carbon utilization in BioLog plates. This is unusual for the bacterium not to utilize any of the carbon substrate provided in BioLog. The BioLog system determines respiratory activity and the tetrazolium dye used to assay the respiratory activity might be potentially toxic to certain organisms. More research is needed for identifying appropriate growth promoting substances before defining the carbon substrate utilization profile of strain B12^T^.

### Chemotaxonomic Characterizations of Strain B12^T^

Cellular fatty acids were analyzed by collecting biomass of a freshly grown culture at optimum growth conditions stated above. The cellular fatty acids were extracted, methylated, and analyzed by gas chromatography, as per the Sherlock Microbial Identification System (MIDI version 4.0) described previously (Muller et al., 1998; Pandey et al., 2002). A combined analysis by gas chromatography coupled to a mass spectrometer was used to confirm the identity of the fatty acids based on retention time and mass spectral data. The position of the double bond was confirmed by a derivatization to the corresponding dimethyl disulfide adduct (Moss and Lambert-Fair, 1989). Whole cell sugars and the peptidoglycan structure were determined according to Schumann (2011). The analyses of respiratory quinones and polar lipids were performed with 200 mg freeze-dried cells, which were previously incubated in glucose yeast extract malt extract medium (GYM) medium at 28°C and harvested in the stationary phase. The extraction of quinones was carried out using the two-stage method (Tindall, 1990a).

Polar lipids were separated by two-dimensional silica gel thin layer chromatography (Macherey-Nagel, 2007). The first direction was developed in chloroform:methanol:water (65:25:4, v/v/v), and the second in chloroform:methanol:acetic acid:water (80:12:15:4, v/v/v/v). Total lipid material was detected using molybdatophosphoric acid and specific functional groups by using spray reagents specific for the groups (Tindall, 1990b).

### Phylogenetics of Strain B12^T^

A loopful of purified B12^T^ culture was subjected to DNA extraction with the Quick DNA Fungal/Bacterial Miniprep kit (Zymo Research, Irvine, CA, United States), as per the manufacturer’s protocol. The extracted DNA was eluted in 50 μL of molecular grade water and stored at −20°C until further analysis. The 16S rRNA gene (Suzuki et al., 2001; Checinska et al., 2015) was amplified using a universal primer set (Yamamoto and Harayama, 1995) as per previously established protocols. The amplified products were treated with Antarctic phosphatase and exonuclease (New England Biolabs, Ipswich, MA, United States) to remove 5′- and 3′-phosphates from unused dNTPs before sequencing. The resulting sequences were assembled using SeqMan Pro from the DNAStar Lasergene package (DNASTAR Inc., Madison, WI, United States). Bacterial sequences were compared with the EzTaxon-e and EzBioCloud databases (Kim et al., 2012; Yoon et al., 2017) and identified based on the closest percentage similarity to previously identified microbial type strains. An alignment of the B12^T^ 16S rRNA Sanger sequence and those collected from 50 members of class *Actinobacteria* found in public database was created using Clustal Omega (v. 1.2.1). A maximum-likelihood phylogeny based on this 16S rRNA gene alignment was generated using FastTree (v. 2.1.10), and bootstrap values were calculated using PHYLIP Seqboot (v. 3.696) and a script provided by the authors of FastTree, CompareToBootstrap.pl. Neighbor-joining and maximum-parsimony phylogenies based on the same 16S rRNA gene alignment were generated using PHYLIP (v. 3.696) and nodes with the same branching pattern in all three algorithms are highlighted in the phylogenetic tree (Figure 2).

### Whole Genome Sequencing of Strain B12^T^

The sequencing and analysis of the WGS were carried out as previously described (Singh et al., 2017) with minor modifications. In brief, sequencing libraries from isolated B12^T^ strain DNA was prepared using the Nextera DNA Library Preparation Kit from Illumina as per the manufacturer’s instructions. Paired-end sequencing (100 bp) was performed on an Illumina HiSeq 2500 instrument. The data was filtered for high-quality vector, and adapter free reads using cutoff read length of 80 bp and quality score of 20 for genome assembly by using the NGS QC Toolkit v2.3 (Patel and Jain, 2012). The high-quality vector filtered reads were then assembled using the SPAdes genome assembler (Nurk et al., 2013) with default parameters. Subsequently, the assembled genome was annotated using Rapid Annotations using Subsystems Technology (RAST) (Aziz et al., 2008), and their quality was assessed using the Quast package (Gurevich et al., 2013). Furthermore, pairwise ANI was calculated using the established algorithm (Nurk et al., 2013) with EzTaxon-e (Kim et al., 2012). Pairwise AAI comparisons were calculated using an established method (Konstantinidis and Tiedje, 2005). Additionally, dDDH analysis was performed using the Genome-to-Genome Distance Calculator 2.0 (GGDC 2.0) (Meier-Kolthoff et al., 2013). A whole-genome alignment was generated using the CLC (v. 20.0.2) whole genome alignment plugin, and a phylogenetic tree was generated from the alignment using FastTree (v. 2.1.10).

### Multi-Locus Sequence Analysis of Strain B12^T^

Multi-locus sequence analysis (MLSA) based phylogenetic affiliation was performed as reported elsewhere to interpret the phylogenetic affiliation of the *Kineosporiaceae* members considered in this study (Gao and Gupta, 2005). Representative genomes of *Angustibacter, Kineococcus*, *Kineosporia, Pseudo kineococcus, Quadrisphaera*, and *Thalassiella* were used to determine the correct phylogenetic position of strain B12^T^. *Nakamurella multipartita* DSM 44233^T^ was included in the phylogeny to serve as the out-group. Members of genus *Kineosporia* and *Thalassiella* were not selected for MLSA due to the absence of full-length genes in the publicly available database. Full-length DNA sequences for housekeeping genes *frr*, *gyrB*, *nusA*, *rplS*, *rpsB*, and *tsf* were retrieved from eight genomes (including strain B12^T^), and an MLSA phylogenetic tree was generated. Gene sequences were individually aligned using Clustal Omega (v. 1.2.1), and concatenated together using a custom Perl script in the order listed. An approximate maximum-likelihood phylogenetic tree was generated from concatenated sequences using FastTree (v. 2.1.10), and bootstrap values were added to the tree using PHYLIP Seqboot (v. 3.696) and the CompareToBootstrap.pl script provided by the authors of FastTree.

## Results and Discussion

### Phenotypic Characteristics

**FIGURE 1:**
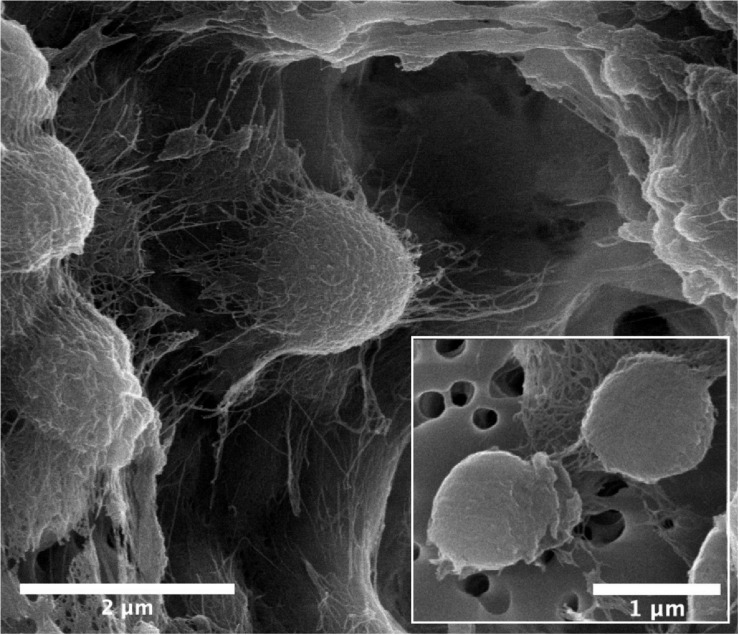
SEM micrographs of *Kineococcus rubinsiae* B12^T^. The compact clustering might be due to the production of extracellular polysaccharide (EPS) seen in abundance (thread-like structures). Upon vigorous agitation the EPS are removed and individual coccoid cells are visualized (inset).

Cells of strain B12^T^ were non-spore forming, Gram-positive, motile, and strictly aerobic. Orange pigmented, round with rough edges and cluster forming colonies (0.6–1.0 mm diameter) were isolated after 72 h of incubation. The optimum growth conditions for the cells of strain B12^T^ were 32°C, 7 to 8 pH, and 0.5% NaCl concentration. Since strain B12^T^ is a slow grower, at least 3–5 days of incubation time was required to see the first visible growth when incubated at the optimum cultural conditions in PTYG medium. Since strain B12^T^ was isolated from chloramphenicol supplemented medium, the purified strain was re-streaked again in PTYG agar medium supplemented with 100 μg chloramphenicol, confirming that strain B12^T^ was resistant to chloramphenicol. The SEM images revealed that the cells of strain B12^T^ are coccoid in shape with 1.0 μm diameter (Figure 1). In general, cells of strain B12^T^ form in clusters due to extracellular polymeric substance (Figure 1), but individual motile cells were also observed.

**FIGURE 2:**
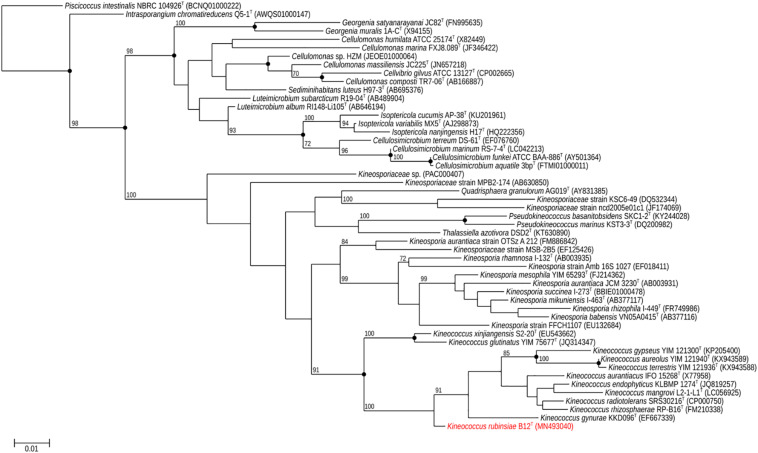
Maximum-likelihood 16S rRNA phylogeny for *Kineococcus rubinsiae* B12^T^ and other members of class *Actinobacteria* reconstructed from 16S rRNA sequences found in public databases. The 16S rRNA sequence from *Piscicoccus intestinalis* NBRC 104926^T^ was used for the out-group. Bootstrapping values are included on internal nodes in the phylogeny, and represent the number of trials (out of 100) that included that particular branching pattern. Strain numbers followed by GenBank accession numbers are included for each sequence in the phylogeny. Black dots on internal nodes represent branch agreement between this maximum-likelihood phylogeny, a neighbor-joining phylogeny, and a maximum-parsimony phylogeny generated from the same sequence alignment.

Comparison of phenotypic characteristics of strain B12^T^ with other members of genus *Kineococcus* are provided in Table 1. Briefly, B12^T^ most closely matches characteristics of its closest neighbor, *K. radiotolerans*, except B12^T^ strain was oxidase positive. Since B12^T^ strain was not able to grow in a minimal media with less than 0.1% glucose or 0.1% yeast extract, phenotypic screening for carbon sources was not successful; instead, genome based phenotypic characterization was performed (Supplementary Table S1). All *Kineococcus* species, including the B12^T^ strain, could be differentiated from *K. radiotolerans* by the utilization of d-fructose and l-alanine as sole carbon and nitrogen sources, respectively. The absence of sucrose utilization by *K. endophytica* and *K. rhizosphaerae* might differentiate them from other members of the *Kineococcus* genera, including the B12^T^ strain. Likewise, d-xylitol assimilation and milk peptonization were observed in *K. glutinatus*, but not in other *Kineococcus* species. L-tryptophan was utilized by most *Kineococcus* species, including strain B12^T^, except by *K. glutinatus* and *K. gypseus*. Gelatin was hydrolyzed by only *K. aureolus* and *K. rhizosphaerae*. *K. aurantiacus* and *K. radiotolerans* were the only members of the genus, including B12^T^, to lack the capability for nitrate reduction. In addition, strain B12^T^ had different characteristic cell wall sugars (Gal, Glu, and Man instead of Ara, and Gal) when compared to other members of the *Kineococcus* genus.

**TABLE 1:** Differential characteristics of strain B12^T^ and type strains of genus *Kineococcus.*

Characteristic	*Strain B12^T^	*K. terrestris* YIM 121936^T^	*K. aureolus* YIM 121940^T^	*K. gypseus* YIM 121300^T^	*K. aurantiacus* JCM 10180^T^	*K. glutinatus* YIM 75677^T^	*K. endophytica* KCTC 19886^T^	*K. rhizosphaerae* KCTC 19366^T^	*K. radiotolerans* DSM 14245^T^
**Growth at/in:**									
5°C	+	+	−	−	−	−	+	+	+
45°C	−	+	+	−	−	+	+	−	−
pH 10.0	−	+	−	+	−	−	−	−	−
pH 5.0	+	+	−	−	−	−	+	−	+
6% NaCl	−	+	+	−	−	−	+	+	−
8% NaCl	−	+	−	−	−	−	+	−	−
Oxidase	+	+	+	−	−	+	+	+	−
DNA G + C content (mol%)	74.2	74.7	75.2	75.1	72.1	74.8	73.4	73.8	74.3
Polar lipids	DPG, PG, PI, PLs	DPG, PG, PGL, PI, PIM, PLs	DPG, PG PGL, PI, PIM, PLs	DPG, PG, PI, PIM, PL	DPG, PG, 2PL	DPG, PG, PI, PIM, PL	DPG, PG, PI, PIM, PL, GL, 3L	DPG, PG, PI, PLs	DPG, PG, PI, PLs
Characteristic sugars	Gal, Glu, Man	Ara, Gal, Glu, Man, Rib	Ara, Gal, Glu, Man, Rib	Ara, Gal, Glu, Man, Rib	Ara, Gal	Glu, Gal, Man, Rib	Ara, Gal	Ara, Gal, Xyl	Ara, Gal

*B12^*T*^ strain phenotypic characteristics were carried out during this study, whereas characteristics of other strains were reproduced from another study (Xu et al., 2017). Differential biochemical characteristics of the new species were not presented because strain B12^T^ would not grow using minimal media. To grow this slow growing strain, either yeast extract or glucose concentration of 0.1% is required. However, B12^T^ strain phenotypic characteristics can be deduced from the genome annotation and pathway analysis (see Supplementary Table S1).

### Chemotaxonomic Characteristics of Strain B12^T^

FAME profiles of strain B12^T^ and related *Kineococcus* genera are shown in Table 2. The majority of the fatty acids produced by strain B12^T^ consisted of anteiso 15:0 (66.3%), similar to other members of *Kineococcus* genera (58–77%). Minor amounts of other fatty acids were detected including anteiso-C_15__:__1_ ω10c (4.6%), iso-C_14__:__0_ (1.0%), iso-C_15__:__0_ (1.1%), and C_14__:__0_ (1.6%). The peak assigned to C_14__:__0_ 2OH by the MIDI system was identified as anteiso-C_15__:__0_ dimethyl acetal (DMA) (2.9%) by GC-MS. Further two peaks that were assigned as C_20__:__4_ (16.4%) and C_17__:__0_ 2OH (2.2%) by the MIDI system, could not be confirmed to represent fatty acid methyl esters by GC-MS and have to be assigned as non-identified hydrophobic compounds. The presence of DMA derivatives in the sample appeared at first sight to be surprising as they are usually only detected in anaerobic bacteria but they show a clear diagnostic fragment at *m*/*z* 75 [(M-OCH_3_)^+^] and a typical [M–31]^+^ fragment in the GC-MS analysis (Maulik et al., 1993; Alves et al., 2013). The presence of significant amounts of branched chain dimethylacetals has been detected in the aerobic taxa *Subtercola boreus* and *Subtercola frigoramans* (Männistö et al., 2000) that was also confirmed by GC-MS. The misidentification of dimethylacetals may be attributed to the fact that the identification of DMA derivatives by the MIDI system is only possible with the use of the ANAEROBE six reference database. This is in contrast to the misidentification of hydroxylated fatty acids as dimethylacetals in members of the *Selenomonadales* (Moore et al., 1994; Helander and Haikara, 1995; Strompl et al., 2000). It is possible that DMA derivatives may be present in other members of genera *Kineococcus* but have been misidentified as hydroxy fatty acids by the MIDI system. The presence of dimethylacetals in the hydrolyzed cellular fatty acid fraction is also indicative of plasmalogens in the polar lipids.

**TABLE 2:** Cellular fatty acid composition of strain B12^T^ and the reference strains.

Fatty acid	Strain B12^T^	*K. terrestris*	*K. aureolus*	*K. gypseus*	*K. aurantiacus*	*K. glutinatus*	*K. endophyticus*	*K. rhizosphaerae*	*K. radiotolerans*
C_12__:__0_	0.7			1.1					
C_14__:__0_ iso	0.8	2.2	2.2		1.9		3.4	1.3	16.5
C_14__:__0_	1.9		1.6	1.5	1.5	2.4	1.6	2.0	
C_14__:__0_ 2-OH							1.9	1.6	
C_15__:__1_ anteiso A	2.3	3.8	2.2	6.9					5.9
Iso-C_15__:__0_						5.4	1.8		
C_15__:__0_ anteiso	63.0	70.8	74.7	58.4	72.8	60.6	76.8	73.8	70.2
C_16__:__0_N alcohol	0.4	1.6	1.2	3.2		1.3	1.1	2.0	1.0
C_16__:__0_ Iso									1.1
C_16__:__0_	3.7	2.6	3.7	6.6	4.1	5.3	1.8	3.9	0.8
C_16__:__0_ 3OH	3.0								
C_17__:__0_ 2OH	2.7	3.7	4.4	3.7	5.1	1.3	2.2	3.3	
C_17__:__0_ 3OH		3.8	2.1	1.8	1.4	1.7			1.3
C_17__:__1_ ω7c									0.7
C_18__:__1_ ω9c	0.8								
C_18__:__0_	2.8	1.7		7.0	4.4	4.2	1.7	4.1	
C_18__:__0_ 3OH						1.3			
C_18__:__3_ ω6c(6,9,12)		1.1							
C_19__:__0_	0.7								
C_19__:__0_ 10-methyl		1.2		1.0					
C_20__:__4_ ω6,9,12,15c	16.2					3.3			
C_20__:__2_ ω6,9c	1.2								
Summed feature 1					1.1				
Summed feature 4		2.7	2.5	5.3	2.1	5.3	2.4	2.5	
Summed feature 7		1.6	1.0	2.1	1.1	1.4	1.0	1.6	

*Values are percentages of total fatty acids. The FAME profiles of the species other than B12^*T*^ strain were taken from Phillips et al. (2002), Li et al. (2015), and Xu et al. (2017).*

The differential characteristics of various genera of the family *Kineosporiaceae* based on chemotaxonomic profiles are shown in Table 3. Most of these chemotaxonomic characteristics are similar to the other members of genus *Kineococcus*. The major menaquinone of strain B12^T^ is MK-9(H2), with minor amounts MK-9 and MK-8(H2) detected (Supplementary Figure S1). Mycolic acids were not detected. The polar lipid analysis of strain B12^T^ showed the presence of diphosphatidylglycerol, phosphatidylglycerol, phosphatidylinositol, a monoacylated dimannosylphosphatdylinositol, several unidentified phosph- olipids, an unidentified glycolipid, and an unidentified lipid (Supplementary Figure S2). The polar lipids may contain plasmalogens. However, the novel strain B12^T^ showed major differences in its cell-wall diamino acid composition with the members of genus *Kineococcus.* The cell wall of strain B12^T^ contain meso-diaminopimelic acid, A1 gamma, and A31, whereas *Kineococcus* species were reported to contain only meso-diaminopimelic acid (Lee, 2006; Tamura et al., 2010). Furthermore, whole cell wall sugars detected for strain B12^T^ are galactose, glucose, and mannose, while other *Kineococcus* species possess galactose, and arabinose. Except a few, there were no major cell-wall and polar-lipid characteristic differences between the *Kineococcus* and related genera as shown in Table 3.

**TABLE 3:** Differential characteristics of strain B12^T^ and related taxa.

Characteristic	Strain B12^T^	*Kineococcus*	*Pseudokineococcus*	*Quadrisphaera*	*Kineosporia*	*Angustibacter*
Cell morphology	Cocci in pairs, tetrads and clusters	Cocci in tetrad arrangements	Cocci singly, in pairs or in clusters	Cocci in tetrad arrangements	Single spores borne at tips of substrate hyphae and spore clusters on a sporophore	Irregular rods and cocci
Motility	Motile	Motile	Motile	Non-motile	Motile	Non-motile
Cell-wall diamino acid(s)	*meso-Dap, A1 gamma, A31*	*meso-*A_2_pm	*meso-*A_2_pm	*meso-*A_2_pm	*meso-* and LL-A_2_pm	*meso-*A_2_pm
Fatty acid type	S, I, A	S, I, A	S, I, A	S, I, A, U	S, U, M	S, I, A, U, M
Predominant menaquinone	MK-9(H_2_)	MK-9(H_2_)	MK-9(H_2_)	MK-8(H_2_)	MK-9(H4)	MK-9(H_4_)
Polar lipids	DPG, PG, PI, PLs	DPG, PG, GL	PG, PI	DPG, PG, PI	PC, DPG, PI, PIM	DPG, PG, PI, PIM
Characteristic sugars	Gal, Glu, Man	Gal, Ara	Gal, Ara	ND	Gal, Glu, Man, Rib	Gal, Glu, Rib
DNA G + C content (mol%)	74.2	73–77	76.6	75	69–71	71

*Data from Lee (2006) and Tamura et al. (2010) and this study. A_2_pm, diaminopimelic acid; A, anteiso-methyl-branched; I, iso-methyl-branched; M, 9- 10-methyl-branched; S, straight-chain saturated; U, monounsaturated; DPG, diphosphatidylglycerol; GL, unknown glycolipid; PC, phosphatidylcholine; PG, phosphatidylglycerol; PI, phosphatidylinositol; PIM, phosphatidylinositol mannosides; PL, unknown phospholipids; PGL, unknown phosphoglycolipid; Ara, arabinose; Gal, galactose; Glu, glucose; Man, mannose; Rha, rhamnose; Rib, ribose; ND, no data.*

### Phylogenomic Characteristics of Strain B12^T^

**FIGURE 3:**
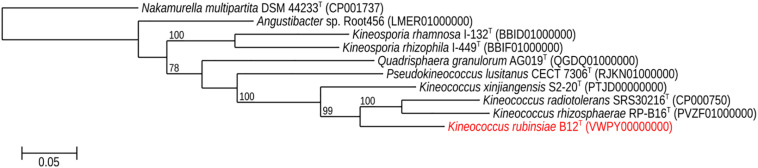
Maximum-likelihood *gyrB* phylogeny for *Kineococcus rubinsiae* B12^T^ and other members of family *Kineosporiaceae* reconstructed from full-length *gyrB* sequences found in public databases. The *gyrB* sequence from *Nakamurella multipartita* DSM 4423^T^ was used for the out-group. Bootstrapping values are included on internal nodes in the phylogeny, and represent the number of trials (out of 100) that included that branching pattern. Strain numbers followed by GenBank accession numbers are included for each sequence in the phylogeny.

The16S rRNA gene sequences of ∼1,000 strains archived from the SAF were queried against the 16S rRNA gene retrieved from the WGS of strain B12^T^. Phylogenetic analysis of the 16S rRNA gene (1,508 bp) indicated that it formed a distinct cluster with members of the genus *Kineococcus* within the radiation tolerant members of the family *Kineosporiaceae*. The 16S rRNA sequence of strain B12^T^ exhibited high sequence similarity with *K. radiotolerans* ATCC BAA 149 (97.9%). Low 16S rRNA gene sequence similarities (93.2–97.9%) with 28 members of the family *Kineosporiaceae* (Figure 2), including *Angustibacter* (93%), *Kineococcus* (96% to 97%), *Kineosporia* (94% to 95%), *Pseudokineococcus* (93%), *Quadrisphaera* (93%), and *Thalassiella* (94%), showed that strain B12^T^ belong to the members of this family. Similarly, when 1-kb *gyrB* sequences were retrieved from WGS of the strain B12^T^ and compared with available *gyrB* sequences (Figure 3) of other members of the family *Kineosporiaceae, K. radiotolerans* ATCC BAA 149 was the closest relative, but the low similarity percentage (85.6%) confirmed that strain B12^T^ is phylogenetically distinct from *K. radiotolerans.*

**FIGURE 4:**
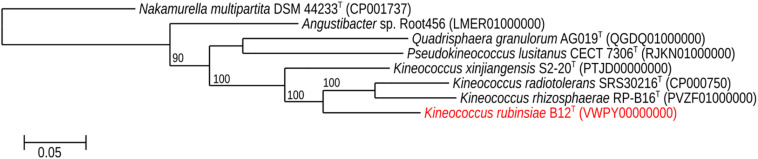
Maximum-likelihood phylogeny for *Kineococcus rubinsiae* B12^T^ and other members of family Kineosporiaceae reconstructed from concatenated, full-length nucleotides sequences of genes *frr*, *gyrB*, *nusA*, *rplS*, *rpsB*, and *tsf*. The concatenated sequence from *Nakamurella multipartita* DSM 4423^T^ was used for the out-group. Bootstrapping values are included on internal nodes in the phylogeny, and represent the number of trials (out of 100) that included that branching pattern. Strain numbers followed by GenBank accession numbers are included for each sequence in the phylogeny.

The MLSA phylogenetic tree with full-length DNA sequences for housekeeping genes *frr*, *gyrB*, *nusA*, *rplS*, *rpsB*, and *tsf* was generated and shown in Figure 4. Genomes belonging to *Angustibacter, Kineococcus*, *Pseudokineococcus*, and *Quadrisphaera* formed a family level clade but showed inter-genus distinction among themselves. Similar to the *gyrB* phylogeny, the MLSA tree clearly showed strain B12^T^ far separated from the type strain of *K. radiotolerans*, but within the *Kineococcus* clade.

### Genome Sequence Characteristics of Strain B12^T^

The Illumina HiSeq 2500 platform yielded 5,435,202 paired-end reads from the sequencing of strain B12^T^. Subsequent trimming and quality filtering of the paired-end sequences resulted in a total of 5,352,711 reads. The final assembled draft genome consists of 119 contigs comprising 4,880,137 bp with an N50 contig length of 66,629 bp. The largest contig assembled accounted for 392,027 bp. The final draft genome has a mean coverage of 100×, and G + C mol% of 74.16%, similar to other members of the *Kineococcus* genera. Table 4 provides a complete summary of genome assembly statistics.

**TABLE 4:** Genome assembly characteristics and quality check.

Attribute	Value
# contigs	119
# contigs (≥0 bp)	119
# contigs (≥1000 bp)	119
# contigs (≥5000 bp)	106
# contigs (≥10000 bp)	90
# contigs (≥25000 bp)	61
# contigs (≥50000 bp)	32
Largest contig	392,027
Total length	4,880,137
Total length (≥0 bp)	4,880,137
Total length (≥1000 bp)	4,880,137
Total length (≥5000 bp)	4,823,868
Total length (≥10000 bp)	4,710,190
Total length (≥25000 bp)	4,229,606
Total length (≥50000 bp)	3,215,408
N50	66,629
N75	35,634
L50	19
L75	43
GC (%)	74.16
# N’s	0
# N’s per 100 kbp	0
Coverage	100×
Coding sequences	4872
Finishing quality	High-quality draft
Library used	Illumina paired-end
Assembler	Spades v3.2

The ANI, AAI, and dDDH values of the strain B12^T^ were compared with the members of the *Kineococcus* genus (Table 5). The closest genomic neighbor to strain B12^T^ was *Kineococcus radiotolerans* GCF_000017305.1, which was evident from the ANI, AAI, and dDNA-DNA hybridization values (Table 5). The ANI values showed 72–78% similarity, and AAI values showed 66–72% with other members of the *Kineococcus* genera, demonstrating that B12^T^ is a novel species within this genus (<95% for ANI and AAI). The dDDH comparison of strain B12^T^ genome with *Kineococcus* genera showed only 20–22% similarity, demonstrating that B12^T^ is a novel species (<70%) that is distantly related to other members of genera *Kineococcus*.

**TABLE 5:** ANI, AAI, and dDDH comparison between strain B12^T^ and phylogenetic neighbors from *Kineococcus*, *Angustibacter, Kineosporia, Nakamurella, Pseudokineococcus*, and *Quadrisphaera* genera.

Reference genomes compared with B12^T^*	GenBank accession	ANI value (%)	AAI value (%)**	dDDH
*Angustibacter* sp. Root456	GCA_001426435.1	72.87	57	19.7
*Kineococcus radiotolerans* ATCC BAA 149^T^	GCF_000017305.1	77.87	72	21.7
*Kineococcus rhizosphaerae* DSM 19711^T^	GCA_003002055.1	76.89	71	21.4
*Kineococcus xinjiangensis* DSM 22857^T^	GCA_002934625.1	75.03	66	20.3
*Kineosporia aurantiaca* JCM 3230^T^	GCA_001315325.1	73.74		22.1
*Kineosporia mikuniensis* JCM 9961^T^	GCA_001315725.1	75.05		24.4
*Kineosporia rhamnosa* JCM 9954^T^	GCA_001315665.1	72.87		21.0
*Kineosporia rhizophila* JCM 9960^T^	GCA_001315705.1	73.01		20.4
*Kineosporia* sp. A 224	GCA_002198655.1	73.27	54	19.7
*Kineosporia* sp. R H 3	GCA_002198675.1	73.21	54	19.5
*Kineosporia succinea* JCM 9957^T^	GCA_001315685.1	75.05		21.4
*Nakamurella multipartita* DSM 44233^T^	GCA_000024365.1	70.23	50	18.3
*Pseudokineococcus lusitanus* CECT 7306^T^	GCA_003751265.1	72.73	58	19.0
*Quadrisphaera granulorum* DSM 44889^T^	GCA_003149145.1	72.39	57	18.9
*Quadrisphaera* sp. DD2A	GCA_008041935.1	72.73	58	18.7
*Quadrisphaera* sp. DSM 44207	GCA_900101335.1	73.42	50	19.4

*Available genomes of nearest neighbors, based on 16S rRNA and *gyr*B gene similarities, were compared. **AAI comparisons not run for phylogenetic neighbors missing amino acid sequences in public databases.

**FIGURE 5:**
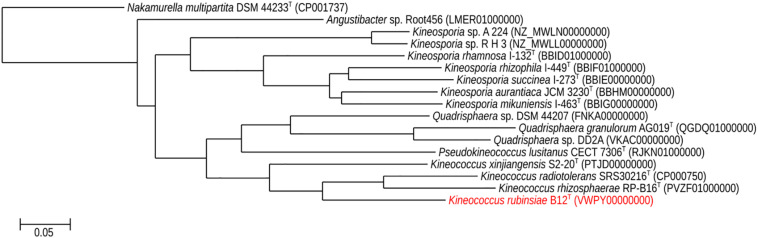
Maximum-likelihood phylogeny for *Kineococcus rubinsiae* B12^T^ and other members of family Kineosporiaceae reconstructed from full-length genome sequences. The genome of *Nakamurella multipartita* DSM 4423^T^ was used for the out-group. Strain numbers followed by GenBank accession numbers are included for each sequence in the phylogeny.

A phylogenetic tree (Figure 5) was generated from a whole-genome alignment of 17 genomes, employing FastTree (v. 2.1.10) and PHYLIP Seqboot (v. 3.696). Even though MLSA clearly placed strain B12^T^ within the *Kineococcus* genus, whole genome phylogenetic analysis was carried out to validate these results using all available WGS of *Kineosporiaceae* reference genomes (*n* = 17) from GenBank. The whole-genome analyses confirmed and gave a strong validation to the MLST/*gyrB* data, confirming that strain B12^T^ is a novel member of a genus *Kineococcus*.

### Functional Gene Properties of Strain B12^T^

The strain B12^T^ genome was analyzed to understand its genetic makeup and its metabolic potential. RAST annotation detected 4,872 coding sequences in the B12^T^ WGS. A major fraction of the annotated genes was comprised of amino acids and derivatives (299), carbohydrate metabolism (246), protein metabolism (207), genes associated with cofactors, vitamins, prosthetic groups, pigments metabolism (141), and DNA (100) and RNA (33) metabolism (Table 6). Genes responsible for motility and chemotaxis (36), metabolism of aromatic compounds (26), and stress response (48) were also observed.

**TABLE 6:** Number and proportion of genes associated with various functions in strain B12^T^.

Functional description	Predicted genes*	Percentage (%)
Cofactors. vitamins. prosthetic groups. pigments	141	8.45
Cell Wall and capsule	24	1.44
Virulence. disease and defense	50	3.00
Potassium metabolism	7	0.42
Miscellaneous	49	2.94
Membrane transport	64	3.83
Iron acquisition and metabolism	4	0.24
RNA Metabolism	33	1.98
Nucleosides and nucleotides	95	5.69
Protein metabolism	207	12.40
Motility and chemotaxis	36	2.16
Regulation and cell signaling	22	1.32
Secondary metabolism	8	0.48
DNA metabolism	100	5.99
Fatty acids. lipids., and isoprenoids	79	4.73
Nitrogen metabolism	13	0.78
Dormancy and sporulation	2	0.12
Respiration	59	3.54
Stress response	48	2.88
Metabolism of aromatic compounds	26	1.56
Amino acids and derivatives	299	17.91
Sulfur metabolism	20	1.20
Phosphorus metabolism	37	2.22
Carbohydrates	246	14.74

*Total protein coding genes as per annotated genome.

A close look at the annotated draft genome (Supplementary Dataset 1) predicts that strain B12^T^ may be highly resistant to osmotic, oxidative, and periplasmic stress, a prime requirement for survival in a SAF-like ultra-low biomass environment regularly cleaned with industrial reagents (La Duc et al., 2012). Strain B12^T^ also harbors the degradation pathway for geraniol, dichlorodiphenyltrichloroethane, chlorocyclohexane, chlorobenzene, benzoate, bisphenol, fluorobenzoate, and furfural, which may provide a pathway for survival from industrial-strength cleaning reagents. *Actinobacteria* have been known to produce antibiotics, and the WGS of the strain B12^T^ also revealed genetic pathways related to the production of these antibiotics. Pathway analysis predict the production of novobiocin, puromycin, tetracycline, penicillin, and cephalosporin from the draft genome. The production of antibiotics would be helpful in reducing competition for B12^T^, and may provide an advantage in the ultralow biomass SAF environment. This makes the organism B12^T^ a potential candidate for industrial use.

### Proposal for a New Species

Strain B12^T^ shares a maximum 97.9% 16S rRNA similarity with *K. radiotolerans*, its closest phylogenetic neighbor within the *Kineosporiaceae* family. These values fall below the 98.7% threshold as demonstrated to delineate bacterial species (Stackebrandt and Ebers, 2006). The phenotypic, phylogenetic, morphological, and genomic characteristics provide evidence to differentiate strain B12^T^ from the members of family *Kineosporiaceae*. Similarly, the ANI, AAI, and dDDH values were lower than the threshold, further corroborating the findings to classify strain B12^T^ as a new species within the family *Kineosporiaceae*. Based on polytaxonomic and WGS analyses, a novel species, *K. rubinsiae* sp. nov., is proposed, with strain B12^T^ (=FJII-L1-CM-PAB2^T^ = DSM 110506^T^) being the type strain of the species *K. rubinsiae*.

### Description of *Kineococcus rubinsiae* sp. nov.

*Kineococcus rubinsiae* sp. nov. (ru.bin.si.ae. N.L. fem. n. *rubinsiae* named in honor of a NASA astronaut (Kate Rubins) who is a molecular microbiologist and the first person to perform DNA sequencing in space).

Cells are Gram-positive cocci that are 0.6–1.0 mm in diameter and occur in tetrads or clusters due to extracellular polysaccharide secretion. Colonies are circular, convex with a diameter of approximately 0.6–1.0 mm, and orange in color after 72 h of incubation on PDA or PTYG medium at 28 to 32°C. A slow growing aerobic bacterium with an optimum temperature of 32°C and incubation period of 3–5 days. Bacteria can exhibit motility after 72 h of growth in a liquid medium. Cells exhibit growth in a cluster formation. The pH tolerance is between 6.0 and 9.0, and shows positive growth at 0–5% NaCl. Cells will not utilize any of the carbon substrates in the BioLog system. Cells are resistant to chloramphenicol and multiply in the PDA supplemented with 100 μg/mL. The predominant fatty acid is anteiso-C_15:0_. Whole cell sugars were galactose, glucose and mannose with minor amounts of arabinose and ribose. The major menaquinone is MK-9(H2) (Supplementary Figure S1). Polar lipids comprise diphosphatidylglycerol, phosphatidylglycerol, phosphatidyl inositol, an unidentified phospholipid, an unidentified glycolipid and an unidentified phosphoglycolipid (Supplementary Figure S2). The DNA G + C content of the type strain is 74.16 mol%.

The type strain, B12^T^ (=FJII-L1-CM-PAB2^T^; NRRL B-65556^T^ = DSM 110506^T^), was isolated from the East side of the JPL-SAF cleanroom where crew enter the room. WGS (VWPY00000000) and 16S rRNA Sanger sequence (MN493040) are available in NCBI GenBank.

### Nucleotide Sequence Deposition

The draft genome sequence of type strain B12^T^ was deposited in NCBI GenBank. The version described in this paper is the first version, and the accession number for the *K. rubinsiae* strain B12^T^ is VWPY00000000. The Sanger sequence of the 16S rRNA gene is deposited in GenBank under accession number MN493040.

## Data Availability Statement

The datasets generated for this study can be found in the NCBI-VWPY00000000.

## Author Contributions

KV, SM, and NS conceived and designed the experiments. SM, NS, JW, and CP performed the experiments. NS analyzed the genomic data analysis inclusive of *de novo* assemblies and verification, scaffold quality assessment, and annotation and generated all the whole genome and protein level alignment for positional description of organism in the tree of life. JW independently verified the genome assembly, generated alignments for all gene trees in the manuscript, and manually curated the tree images. SM isolated the type strain, carried out the phenotypic assays, FAME, and biochemical characterization. CP conducted SEM related tests and imaged the microscopic characteristics of the strain. KV compiled the contribution of write-ups from all authors associated with phenotype (SM), genotype and table generation (NS), MLSA and figure generation (JW), as well as SEM images (CP). BT, SV, RP, and MN-S conducted the chemotaxonomic analysis. All authors read and approved the final manuscript.

## Conflict of Interest

The authors declare that the research was conducted in the absence of any commercial or financial relationships that could be construed as a potential conflict of interest.

## Supplementary Material

FIGURE S1 Quinone determination of *Kineococcus rubinsiae* B12^T^.

FIGURE S2 Lipid profiles of *Kineococcus rubinsiae* B12^T^.
