# Supplementary material for: Description of Chloramphenicol Resistant Kineococcus rubinsiae sp. nov. Isolated From a Spacecraft Assembly Facility
Source: Front Microbiol. 2020 Aug 18;11:1957. doi: 10.3389/fmicb.2020.01957 (PMC7472656; doi:10.3389/fmicb.2020.01957)
Supplement: FIGURE S1 — Quinone determination of Kineococcus rubinsiae B12T. [file Image_1.pdf]

Quinone Determination

DSMZ

**Data file:**	DSM 11050620200218 112116Chinone_spektra_2mm_HPLC2.amx.dx		
**Sequence Name:**	200218_afr	**Processing method:**	190304_Chinone_Standards7.pmx
**Sample name:**	DSM 110506	**Manually modified:**	Manual Integration
**Instrument:**	HPLC2	**Injection date:**	2020-02-18 11:22:11+01:00
**Inj. volume:**	5.0	**Location:**	P1-A3
**Acq. method:**	Chinone_spektra_2mm_HPLC2.<br>amx		

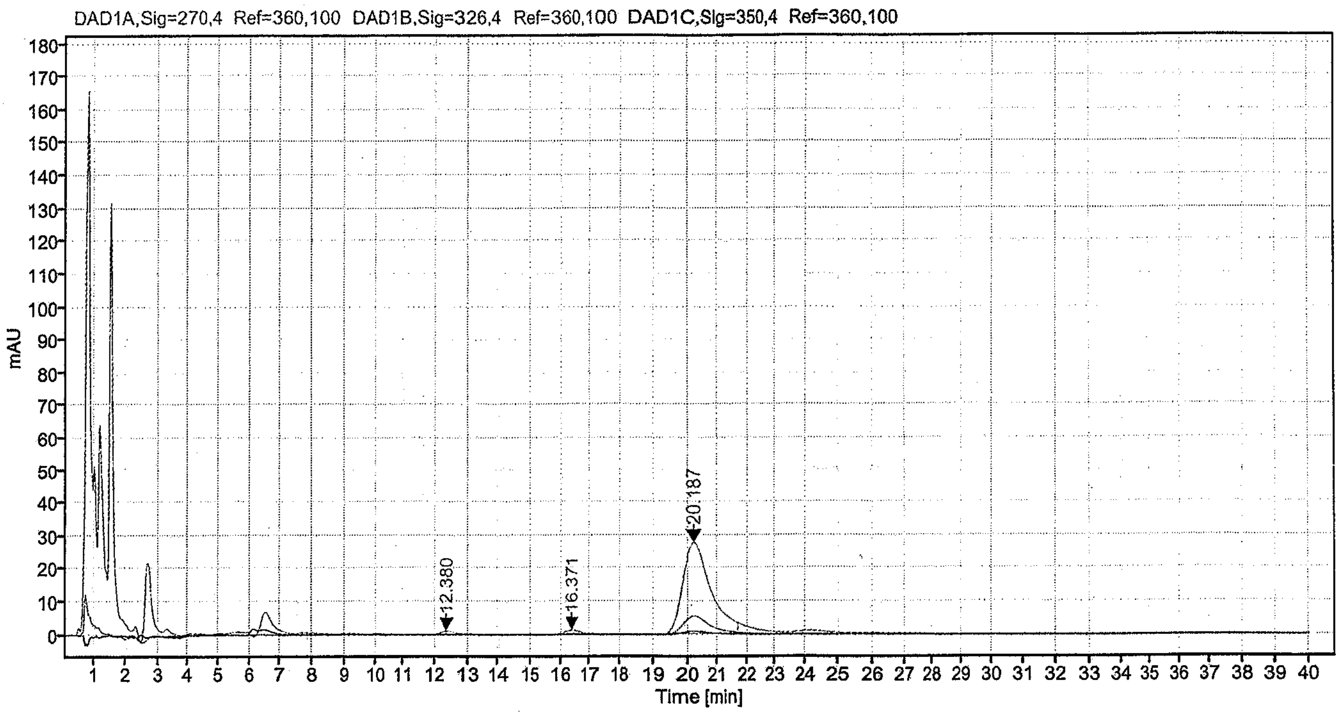

Signal: DAD1A,Sig=270,4 Ref=360,100

RT [min]	Type	Width [min]	Area	Height	Area%	Name
12.380	MM m	0.44	30.37	1.05	1.74	MK 8 H2
16.371	MM m	0.54	45.58	1.22	2.61	MK 9
20.187	MM m	0.89	1673.50	27.69	95.66	MK 9 H2
		Sum	1749.45			
