# Supplementary material for: Description of Chloramphenicol Resistant Kineococcus rubinsiae sp. nov. Isolated From a Spacecraft Assembly Facility
Source: Front Microbiol. 2020 Aug 18;11:1957. doi: 10.3389/fmicb.2020.01957 (PMC7472656; doi:10.3389/fmicb.2020.01957)
Supplement: FIGURE S2 — Lipid profiles of Kineococcus rubinsiae B12T. [file Image_2.pdf]

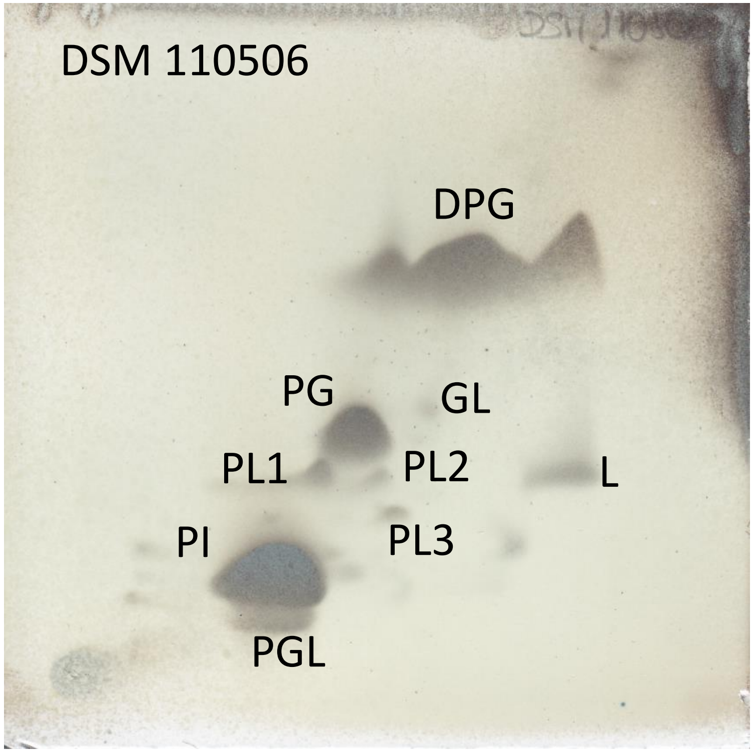

PG = phosphatidylglycerol
DPG = diphosphatidylglycerol
PI = phosphatdylinositol
PL1 – PL3 = phospholipids
L = lipid
GL = glycolipid
PGL = phosphoglycolipid with a Rf value consistent with this being a monoacylated dimannosylphosphatdylinositol
